# Polarization-sensitive intensity diffraction tomography

**DOI:** 10.1038/s41377-023-01151-0

**Published:** 2023-05-18

**Authors:** Seungri Song, Jeongsoo Kim, Taegyun Moon, Baekcheon Seong, Woovin Kim, Chang-Hyuk Yoo, Jun-Kyu Choi, Chulmin Joo

**Affiliations:** 1 Department of Mechanical Engineering, Yonsei University, Seoul, 03722 Republic of Korea; 2 Small Machines Company, Ltd., Seoul, 04808 Republic of Korea

**Keywords:** Polarization microscopy, Biophotonics

## Abstract

Optical anisotropy, which is an intrinsic property of many materials, originates from the structural arrangement of molecular structures, and to date, various polarization-sensitive imaging (PSI) methods have been developed to investigate the nature of anisotropic materials. In particular, the recently developed tomographic PSI technologies enable the investigation of anisotropic materials through volumetric mappings of the anisotropy distribution of these materials. However, these reported methods mostly operate on a single scattering model, and are thus not suitable for three-dimensional (3D) PSI imaging of multiple scattering samples. Here, we present a novel reference-free 3D polarization-sensitive computational imaging technique—polarization-sensitive intensity diffraction tomography (PS-IDT)—that enables the reconstruction of 3D anisotropy distribution of both weakly and multiple scattering specimens from multiple intensity-only measurements. A 3D anisotropic object is illuminated by circularly polarized plane waves at various illumination angles to encode the isotropic and anisotropic structural information into 2D intensity information. These information are then recorded separately through two orthogonal analyzer states, and a 3D Jones matrix is iteratively reconstructed based on the vectorial multi-slice beam propagation model and gradient descent method. We demonstrate the 3D anisotropy imaging capabilities of PS-IDT by presenting 3D anisotropy maps of various samples, including potato starch granules and tardigrade.

## Abstract (web summary)

The three-dimensional (3D) distribution of isotropic and anisotropic properties of multi-scattering objects can be effectively encoded into multiple intensity images at different input polarization states and illumination angles. Polarization-sensitive intensity diffraction tomography (PS-IDT) has demonstrated the tomographic reconstruction of 3D multiple scattering objects with fully or partially distributed anisotropy structures using vectorial multi-slice beam propagation forward model and optimization approach from captured phaseless measurements.

## Graphical abstract

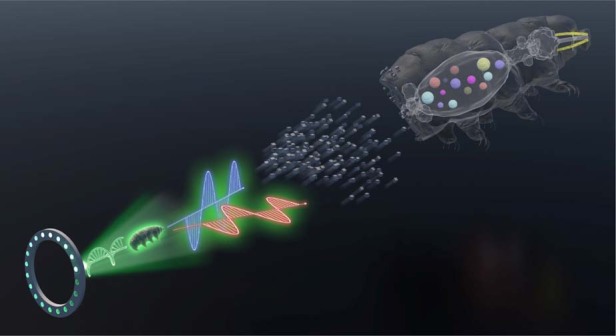

## Introduction

Over the past decade, significant efforts have been devoted to the development of a variety of computational imaging modalities for structural and molecular interpretation of micro-scale transparent specimens^1^. Among the numerous achievements, computational quantitative phase imaging (QPI) techniques have emerged as innovative label-free imaging methods that can not only reconstruct complex object fields, i.e., amplitude and phase delay or refractive indices of two-dimensional^2–10^ (2D) and three-dimensional^11–19^ (3D) transparent objects, but also overcome some of the limitations exhibited by the conventional imaging methods (e.g., limited space-bandwidth product, etc.). With the remarkable advances in phase retrieval algorithms and computing hardware, computational microscopy is now widely applied in biomedicine, chemistry, materials science, and various other disciplines^5,20^. However, these imaging techniques are based on the scalar Helmholtz equations, and therefore, do not provide accurate information on the intrinsic properties of optically anisotropic materials.

Recently, diverse quantitative polarization-sensitive imaging (QPSI) systems have been developed to explore the nature of optically anisotropic materials. Various forms of digital holography-based QPSI techniques have been introduced based on off-axis interferometry^21–26^ and in-line holography^27,28^, and demonstrated their potentials in material characterization and disease diagnosis. Non-interferometric QPSI methods have also been developed based on, for examples, differential phase contrast microscopy, Fourier ptychography, ptychography, lensless imaging, and single-pixel imaging with their unique advantages^29–37^. However, these QPSI methods provide only the 2D projection information that conceal the 3D anisotropy distributions. The recently developed interferometric and non-interferometric configurations of polarization-sensitive (PS) tomography have demonstrated the reconstruction of 3D anisotropy distributions of various specimens^38–41^. However, interferometric approaches^38,41^ have inherent drawbacks, such as phase instability, speckle noise, and complex alignment, whereas the non-interferometric approaches^39,40^ require mechanical sample/objective scanning along the z-direction to reconstruct the 3D anisotropy information. In addition, the reconstruction model of the reported 3D-QPSI methods relies on the weak scattering approximation, and thus, relatively complex structures, such as multicellular organisms, which are optically transparent but exhibit multiple scattering, may not be analyzed by these techniques.

Here, we introduce a novel computational 3D imaging technology, namely polarization-sensitive intensity diffraction tomography (PS-IDT), that enables 3D Jones matrix imaging of multiple-scattering objects using intensity-only measurements based on vectorial multi-slice beam propagation (MSBP) model. For the 2D intensity measurements, an object is illuminated at multiple angles and recorded with four different polarizer/analyzer configurations to encode the 3D structure and anisotropy information. To reconstruct the 3D anisotropy map, we introduce a novel reconstruction framework that accounts for the vectorial nature of polarization by re-formulating the scalar MSBP model^13,42^ and gradient descent method in the vectorial form. PS-IDT enables 3D-QPSI imaging with only a simple modification of conventional polarization light microscope, i.e., replacement of its light source with a ring light-emitting diode (LED) array. We demonstrate the 3D-QPSI capability of PS-IDT by presenting the 3D anisotropy images of a digital phantom and various birefringent samples, including potato starch granules and tardigrade.

## Results

### Vectorial forward model and inverse problem formulation of PS-IDT

First, we introduce the theoretical formulation of PS-IDT to understand the propagation of polarized light through an anisotropic object, and the resultant image formation. The light propagation inside the object is modeled with a vectorial MSBP forward model, and based on the forward model, an inverse problem for the 3D Jones matrix is derived.

**Fig. 1:**
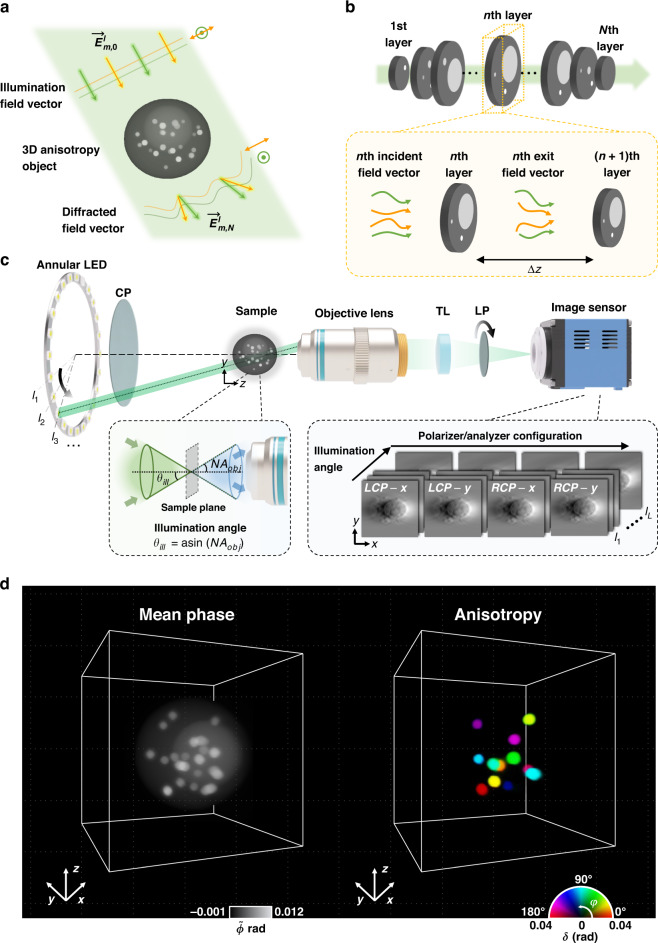
PS-IDT operation principle and experimental setup. **a** A 3D anisotropy object is illuminated by polarized plane waves at various illumination angles, and the resultant diffracted field vector, $\vec E_{m,N}^\ell$, through the object is recorded and processed to obtain 3D Jones matrix of the object. **b** In vectorial MSBP, a 3D anisotropy object is considered as a series of *N* anisotropic thin layers, and the interaction of light with each layer is modeled by considering the propagation of the light through a distance Δ*z* after multiplying the incident field vector by the Jones matrix of the layer. **c** Schematic and measurement processes of PS-IDT. A ring LED array illuminates the object, and a conventional 4-f imaging system collects the diffracted field and forms an image at the sensor plane. The input and output polarization states are modulated by the circular (CP) and linear (LP) polarizers, respectively. TL: tube lens. **d** Exemplary PS-IDT imaging results for a digital phantom. Left and right present the 3D isotropy (mean phase delay $\tilde \phi$) and anisotropy (retardance *δ* and in-plane (xy-plane) optic-axis orientation *φ*) distributions. Definition of mean phase delay is provided in “Methods”

Consider a 3D anisotropy object illuminated by a polarized plane wave at an incidence angle, i.e., $\vec E_{m,0}^\ell \left( {{{\boldsymbol{r}}}} \right)$, where ***r*** denotes the 2D spatial coordinates, and *m* and $\ell$ represent the indices for the input polarization state and illumination angle, respectively (Fig. 1a). In the vectorial MSBP, a 3D object is considered as a series of *N* equally-spaced optically-anisotropic thin layers separated by a constant distance Δ*z*, and the light propagation through the sample is modeled by considering a sequential layer-to-layer propagation of the electric field (Fig. 1b). Using the Jones formalism^43^, an electric field vector of the *n*th layer, $\vec E_n$, is described as:

1 $$\vec E_{m,n}^\ell \left( {{{\boldsymbol{r}}}} \right) = \bar H_{{\varDelta}z}\bar O_n\left( {{{\boldsymbol{r}}}} \right)\vec E_{m,n - 1}^\ell \left( {{{\boldsymbol{r}}}} \right)$$

where $\bar O_n\left( {{{\boldsymbol{r}}}} \right)$ is a 2 × 2 Jones matrix of the *n*th layer, defined as $$\bar O_n\left( {{{\boldsymbol{r}}}} \right) = \left[ {\begin{array}{*{20}{c}} {O_{n,xx}\left( {{{\boldsymbol{r}}}} \right)} & {O_{n,xy}\left( {{{\boldsymbol{r}}}} \right)} \\ {O_{n,yx}\left( {{{\boldsymbol{r}}}} \right)} & {O_{n,yy}\left( {{{\boldsymbol{r}}}} \right)} \end{array}} \right]$$. Note that each layer is considered as a thin non-depolarizing transparent layer, which is a widely used assumption in QPSI^30,36,44–46^. The propagation operation matrix with distance ∆*z*, $\bar H_{{\Delta}z}$, is expressed as $\bar H_{{\Delta}z} = \bar F^{\dagger} h_{{\Delta}z}\left( {{{\boldsymbol{k}}}} \right)\bar F$, where $\bar F$ and $\bar F^{\dagger}$ are the 2D discrete Fourier and inverse Fourier transformation operator matrices, respectively, and ***k*** denotes the 2D spatial frequency coordinates. Here, the superscript † denotes the complex-conjugate transpose, and *h*_Δ*z*_ represents the 2D angular spectrum propagator expressed as $h_{{\varDelta}z}\left( {{{\boldsymbol{k}}}} \right) = \exp \left\{ {j{\varDelta}z\sqrt {\left( {{\textstyle{{2\pi } \over \lambda }}n_{media}} \right)^2 - \left| {{{\boldsymbol{k}}}} \right|^2} } \right\}$, where *n*_*media*_, *λ*, and *j* are the refractive index of the surrounding medium, wavelength of light, and imaginary unit, respectively. Then, the electric field vector at the last layer can be compactly written as:

2 $$\vec E_{m,N}^\ell \left( {{{\boldsymbol{r}}}} \right) = \left[ {\mathop {\prod}\limits_{n = 1}^N {\bar H_{{\varDelta}z}\bar O_n\left( {{{\boldsymbol{r}}}} \right)} } \right]\vec E_{m,0}^\ell \left( {{{\boldsymbol{r}}}} \right)$$

The exit field vector from the 3D object, $\vec E_{m,N}^\ell \left( {{{\boldsymbol{r}}}} \right)$, is collected by the objective lens, and the image is formed on the image sensor passing through the tube lens (Fig. 1c). The final electric field vector at the image plane can then be written as:

3 $$\vec E_m^\ell \left( {{{\boldsymbol{r}}}} \right) = \bar F^{\dagger} \bar P\left( {{{\boldsymbol{k}}}} \right)\bar F\bar H_{ - N{\Delta}z/2}\vec E_{m,N}^\ell \left( {{{\boldsymbol{r}}}} \right)$$

where $\bar P\left( {{{\boldsymbol{k}}}} \right)$ is the aberration-free polarization-independent pupil matrix expressed as $$\bar P\left( {{{\boldsymbol{k}}}} \right) = \left[ {\begin{array}{*{20}{c}} {P\left( {{{\boldsymbol{k}}}} \right)} & 0 \\ 0 & {P\left( {{{\boldsymbol{k}}}} \right)} \end{array}} \right]$$. Note that in Eq. (3), the operator $\bar H_{ - N{\Delta}z/2}$ is applied by assuming that the focal plane is located at the center of the 3D object. The intensity vector of the measurement is $\vec I_m^\ell \left( {{{\boldsymbol{r}}}} \right) = \vec E_m^{\ell ,^\ast }\left( {{{\boldsymbol{r}}}} \right) \odot \vec E_m^\ell \left( {{{\boldsymbol{r}}}} \right)$, where $\odot$ is the Hadamard product, and the superscript * denotes the complex conjugate operator. Each element in the intensity vector is acquired with two orthogonal analyzer states, and the detailed acquisition procedure is described in Methods sub-section “Data acquisition”.

With given input field and the measured intensity vectors, the 3D sample Jones matrix is recovered through an optimization scheme. The reconstruction task is formulated as the minimization problem given by:

4 $$\bar O\left( {{{{\boldsymbol{r}}}}_{3D}} \right) = \mathop {{{{{\mathrm{argmin}}}}}}\limits_{\bar O\left( {{{{\boldsymbol{r}}}}_{3D}} \right)} \left[ {\boldsymbol{\mathcal{D}}\left\{ {\bar O\left( {{{{\boldsymbol{r}}}}_{3D}} \right)} \right\} + \tau {\boldsymbol{\mathcal{R}}}\left\{ {\bar O\left( {{{{\boldsymbol{r}}}}_{3D}} \right)} \right\}} \right]$$

where ${\boldsymbol{\mathcal{D}}}$ is the data fidelity term, ${\boldsymbol{\mathcal{R}}}$ is the regularization term, and *τ* is the regularization parameter, which controls the amount of regularization. The data fidelity term is formulated as the $\ell _2$-norm between the square root of the measured intensity vectors and the estimated amplitude vectors through the forward model computed over the entire illumination conditions:

5 $${\boldsymbol{\mathcal{D}}}\left\{ {\bar O\left( {{{{\boldsymbol{r}}}}_{3D}} \right)} \right\} \buildrel \Delta \over = \mathop {\sum}\limits_{m = 1}^M {\mathop {\sum}\limits_{\ell = 1}^L {\left\| {\sqrt {\vec I_m^\ell \left( {{{\boldsymbol{r}}}} \right)} - \left| {\boldsymbol{\mathcal{G}}_m^\ell \left\{ {\bar O\left( {{{{\boldsymbol{r}}}}_{3D}} \right)} \right\}} \right|} \right\|_2^2} }$$

where *L* and *M* denote the number of illumination angles and input polarization states, respectively; ***r***_3*D*_ is a 3D spatial coordinate, and ${\boldsymbol{\mathcal{G}}}_m^\ell \left\{ \cdot \right\}$ denotes the ${\mathbb{C}}^3 \to {\mathbb{C}}^2$ nonlinear operator of the vectorial MSBP forward model with the $\ell {{\rm{th}}}$ illumination angle and *m*th input polarization state. Note that modulus in Eq. 5 is element-wise operation. For the regularization term, we implement the 3D total variation^47^:

6 $${\boldsymbol{\mathcal{R}}}\left\{ {\bar O\left( {{{{\boldsymbol{r}}}}_{3D}} \right)} \right\} \buildrel \Delta \over = \mathop {\sum}\limits_{\begin{array}{*{20}{c}} {k = } \\ {xx,yx,xy,yy} \end{array}} {\sqrt {\left\{ {\nabla _xO_k\left( {{{{\boldsymbol{r}}}}_{3D}} \right)} \right\}^2 + \left\{ {\nabla _yO_k\left( {{{{\boldsymbol{r}}}}_{3D}} \right)} \right\}^2 + \left\{ {\nabla _zO_k\left( {{{{\boldsymbol{r}}}}_{3D}} \right)} \right\}^2} }$$

where ∇_*x*_, ∇_*y*_, and ∇_*y*_ denote the finite difference operations along the *x*, *y*, and *z* directions, respectively.

### Reconstruction of 3D anisotropic structures

To validate the 3D polarization imaging capability of PS-IDT, we first numerically reconstructed the 3D anisotropy maps of a digital phantom. We considered a multiple-scattering ellipsoidal anisotropic cell with an internal structure compartmentalized with a large nucleus and multiple granules of different sizes and refractive indices. The body of the phantom was set to be optically isotropic with refractive index of 1.34. The nucleic structure was optically anisotropic with refractive indices of the fast and slow axes of 1.36 and 1.38, respectively, and its optic-axis orientation varies in both lateral and axial directions. Ten isotropic and ten anisotropic vesicles were then randomly distributed inside the isotropic body and anisotropic nucleus. The refractive index of the isotropic vesicles was 1.42, and those of the fast and slow axes of the anisotropic vesicles were 1.42 and 1.45, respectively. The surrounding medium had a refractive index of 1.33. Notably, we generated the multiple scattering effect of the sample by designing a relatively larger refractive index difference. The operating conditions for the simulation were identical to that of the experimental setup.

**Fig. 2:**
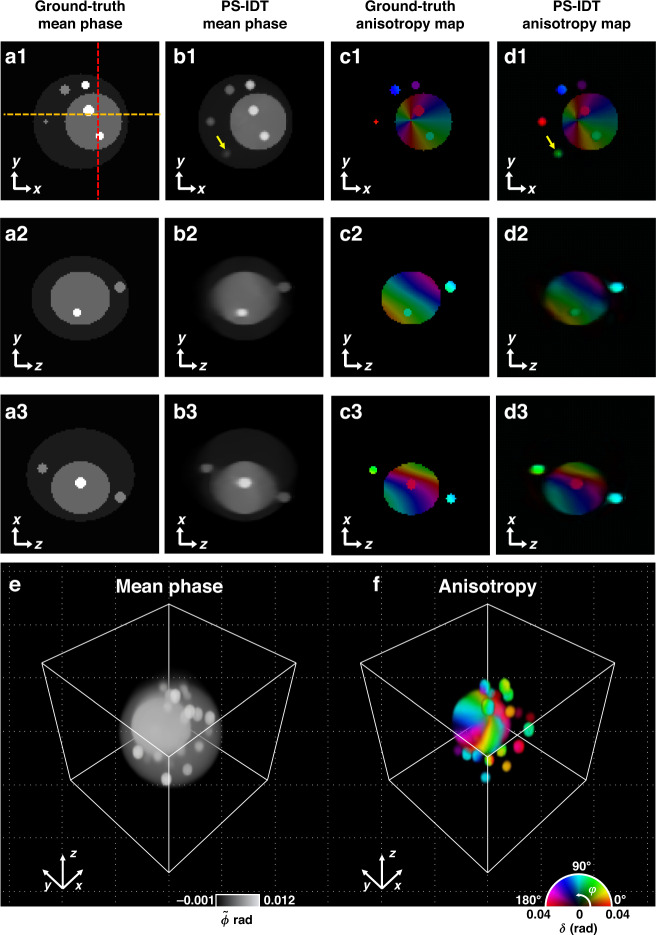
PS-IDT numerical simulation for a 3D multiple-scattering anisotropy phantom. **a1**–**3**, **c1**–**3** Lateral and axial cross-sectional information of mean phase and anisotropy of a digital phantom. **b1**–**3**, **d1**–**3** Corresponding the mean phase and anisotropy images reconstructed by PS-IDT. The first row presents lateral (xy-plane) cross-sectional maps at the center of the 3D phantom, and the second and third rows are the information in the yz and xz sections through the red and orange dashed lines in (**a1**), respectively. **e**, **f** 3D perspectives of the PS-IDT-reconstructed mean phase and anisotropy tomograms

Figures 2a, c show the ground-truth lateral and axial cross-sectional maps of the mean phase and anisotropy of the digital phantom, and Figs. 2b, d are the corresponding images reconstructed with PS-IDT. The origin (x = 0 μm, y = 0 μm, and z = 0 μm) of the coordinate system is defined at the center of the reconstructed volume. The images in the first row in Fig. 2a–d show the information in the xy-plane (z = 0 μm) at the center of the 3D phantom, and the second and third rows correspond to the yz (x = 1.75 μm) and xz (y = 0.65 μm) cross-sectional images through the red and orange dashed lines in Fig. 2a1. The reconstructed mean phase and anisotropy distributions agree well with the ground-truth information, while the features are blurred in the reconstructed images because of the limited resolution (NA = 0.6). It is clearly seen that the PS-IDT successfully reconstructed not only the complex anisotropic nucleic structure but also the particles randomly distributed in both isotropic body and anisotropic nucleus. Note that the unwanted signal, indicated by the yellow arrows in Fig. 2b1, d1, comes from particles in adjacent layers due to the relatively low axial resolution of the angular scanning diffraction tomography technique. The 3D-rendered mean phase and anisotropy tomograms of the phantom are shown in Fig. 2e, f, demonstrating the 3D PSI capability of the PS-IDT technique in the multiple-scattering regime. This 3D-rendered view was obtained using the Amira imaging software (Amira3D pro). A video clip of the 3D rendering results is provided in Supplementary Video 1. We also numerically performed the PS-IDT imaging of liquid crystal sphere as a weakly scattering anisotropy object, and the results are described in section 2 of the Supplementary Information and Supplementary Video 2.

Next, we performed the PS-IDT imaging of a 5 μm polystyrene bead and aggregated potato starch granules as isotropic and multiple-scattering anisotropic objects, respectively. For the experiment, the polystyrene bead and potato starch granules of various sizes were suspended in an index-matching oil ($n_{oil} = 1.516$) and placed between glass coverslips. The sample was illuminated by plane waves from 24 different angles, and the polarization states of the illuminated plane waves were sequentially modulated to be left- and right-handed circularly polarized. The images were obtained through two orthogonal analyzer states, and then used to produce the 3D Jones matrix map. We set Δ*z* = 1 μm over a depth range of −30 μm to 30 μm. The regularization parameter of 1 × 10^−4^ was used for the image reconstruction.

**Fig. 3:**
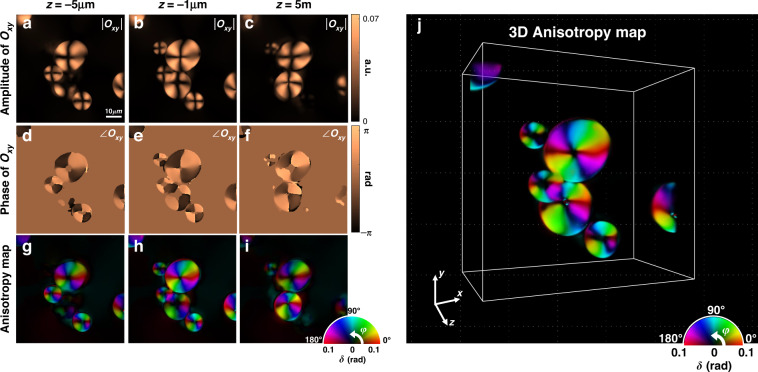
Experimental reconstruction of the 3D anisotropy maps of aggregated potato starch granules. **a**–**f** Amplitude and phase maps of the reconstructed Jones matrix element *O*_*xy*_ of potato starch granules at different depths. **g**–**i** Anisotropy maps extracted from the reconstructed 3D Jones matrix of potato starches. **j** 3D anisotropy map of potato starch granules over a volume of 50 μm × 50 μm × 40 μm

In the case of the polystyrene beads (Fig. S2), one can note that the isotropic information (i.e., mean phase) was well recovered, whereas the anisotropic information was obtained only at the edge of the bead due to edge birefringence^48^. Details of the PS-IDT reconstruction of the polystyrene bead are provided in Supplementary Information (Section 3). In contrast, a potato starch granule features an alternating layered shell structure with amorphous and crystalline regions composed of polymer chains^49^. Thus, this granule exhibits a strong birefringence because of the high degree of radial orientation of the amylopectin crystallites^50,51^. Figure 3a–i shows the Jones matrix and the corresponding anisotropy maps of the potato starch granules obtained at different depths. The measured off-diagonal terms (*O*_*xy*_) at each depth indicate the measured anisotropy properties of the potato starch granules. However, the anisotropic properties, such as linear retardance and optic-axis orientation, are coupled to the elements of the object Jones matrix, and thus, a quantitative interpretation of these properties from the Jones matrix images may be elusive. Therefore, we performed eigenvalue decomposition of the Jones matrix to obtain the linear retardance and identify the optic-axis orientation of the potato starch granules (see “Methods”). Figure 3g–i presents the visualization results of the anisotropic properties (i.e., linear retardance and azimuth optic-axis orientation) using pseudo colormaps. It is clearly seen that the PS-IDT could map out the well-known radially oriented optic-axis distribution and anisotropy information of the aggregated starches, but, due to the limited axial resolution and the use of paraxial BPM model, the starch particles could not be clearly resolved for the particles separated by smaller than 3 μm in axial direction. Recently, split-step non-paraxial (SSNP) method^52,53^ has been introduced as the forward model for multiple-scattering objects. The implementation of the SSNP model in the PT-IDT platform is expected to improve the reconstruction performance for complicated multiple scattering samples. The rendered 3D volumetric anisotropy map of the potato starch granules is shown in Fig. 3j and Supplementary Video 3, demonstrating that the PS-IDT technique can map the 3D anisotropy information from the intensity-only measurements.

To further demonstrate the 3D anisotropy imaging capability of PS-IDT for multiple-scattering samples, we performed PS-IDT imaging of a tardigrade. Tardigrades, also known as water bears, with lengths and heights of less than 0.5 and 0.1 mm, respectively, have various organs, such as cuticles, brains, muscles, claws, guts, and stylets^54^. Due to the optically thick and relatively complex organ distribution, tardigrades are transparent but exhibit multiple scattering characteristics, making it difficult to visualize their structures clearly with a conventional microscope. In addition, it is well-known that some organs, such as the stylet in the buccal apparatus and granules in the midguts, exhibit birefingence^55,56^. The stylet is composed of calcite with a hard structure to penetrate food^57^, and the granules containing birefringent minerals are produced in the midgut as digestive products through the tryptophan catabolism^55,58^. Because of these optical characteristics, a tardigrade was chosen as a suitable sample for demonstrating the imaging capabilities of PS-IDT for transparent multiple-scattering anisotropy objects.

**Fig. 4:**
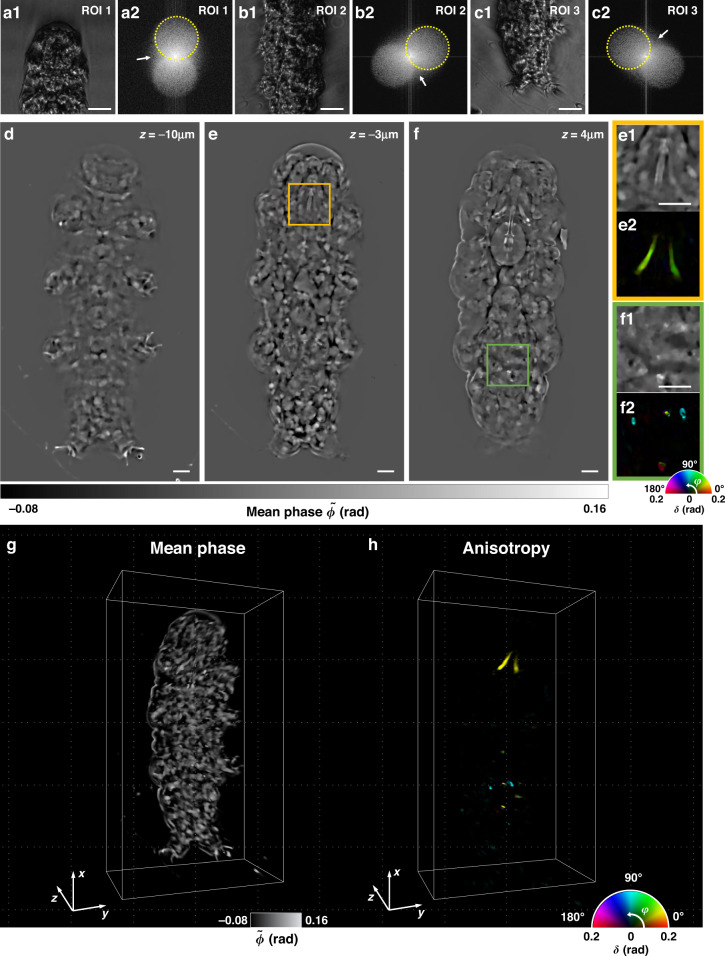
Experimental reconstruction of the 3D anisotropy map of a tardigrade. **a1**–**c1** Representative PS-IDT images of a tardigrade at three different ROIs. **a2–c2** Fourier magnitudes of the measurement results in (**a1**–**c1**). Information outside the pupil (white arrows) in Fourier magnitude is an evidence of the multiple-scattering characteristic of the tardigrade. **d**–**f** Mean phase ($\tilde \phi$) images extracted from the reconstructed 3D Jones matrix of whole tardigrade at different depths of −10, −3, and 4 μm, respectively. **e1–2** and **f1–2** show the magnified mean phase and anisotropy images indicated by the orange and green square boxes in (**e**) and (**f**). **g**, **h** 3D rendering of the mean phase and anisotropy maps of the whole tardigrade over a volume of 150 μm × 80 μm × 60 μm. Scale bars: 10 μm

Because a tardigrade is larger than the field-of-view (FOV) of the PS-IDT, the whole tardigrade image was obtained through 3D stitching using standard 3D rigid-body registration algorithms^59^ after performing the PS-IDT reconstruction on each region of interest (ROI) (80 μm × 80 μm). Figures 4a1–c1 and a2–c2 show the representative intensity measurements and the corresponding Fourier magnitudes of the tardigrade at three different ROIs. As evident from Fig. 4a2–c2, the pupil shifts (yellow dashed line) due to oblique illumination. However, unlike the spectra commonly observed in weakly scattering samples, in this case, we obtain the spatial frequency information outside the pupil (white arrow). These images validate the multiple-scattering characteristics of tardigrades^13^.

Figure 4d–f shows the PS-IDT mean phase images of the whole tardigrade at depths of −10, −3, and 4 μm, respectively. Because the anisotropy information is distributed only in small regions such as the mouse and mid-gut, most of the information of the tardigrade structure can be visualized in the mean phase term. It can be seen that the 3D structures of many organs, such as claws (Fig. 4d), stylet (Fig. 4e), and gut (Fig. 4e, f), are clearly visualized over the entire volume. The PS-IDT images of various other major structures of a tardigrade are provided in Supplementary section 4. Figures 4e1–2 and f1–2 show the enlarged mean phase and anisotropy images that correspond to the regions marked with orange and green square boxes in Fig. 4e, f, respectively. One can see that the optically anisotropic stylet (Fig. 4e2) and granules (Fig. 4f2) are clearly visualized via the anisotropy-based information, while the overall structure of the tardigrade can be observed from the mean phase information. The 3D mean phase delay and anisotropy tomograms of the whole tardigrade are shown in Fig. 4g, h, demonstrating the 3D anisotropy imaging capability of PS-IDT for optically transparent multiple-scattering samples. The depth scanning results and 3D rendering movie clips of the reconstructed 3D mean phase and anisotropy are provided in Supplementary Videos 4 and 5. In particular, we could selectively visualize the 3D structure of the stylet in polarization-based contrast (Supplementary Video 5), which is in good agreement with the structural information observed in scanning electron microscopy^57^. These results clearly indicate that PS-IDT is capable of recovering the 3D anisotropy/isotropic information by taking into account the multiple-scattering characteristic of the sample, even when the anisotropy information is hidden inside the complex isotropic structures of the sample.

## Discussion

In summary, we presented a novel form of computational 3D polarization microscopy that can quantitatively reconstruct the Jones matrix of a 3D anisotropic object based on the vectorial MSBP method and gradient-descent-based optimization strategy. The proposed PS-IDT prototype features reference-free and inertia-free operation, with a lateral resolution of 0.54 μm and an axial resolution of 2.89 μm across an FOV of 80 μm × 80 μm. We developed the vectorial extension of the MSBP forward model to recover 3D Jones matrix of multiple-scattering anisotropic object, and successfully reconstructed its 3D anisotropy information, overcoming the challenges of other 3D PSI techniques based on the single-scattering model (i.e., Rytov and Born)^60^. Our prototype can reconstruct a 3D Jones matrix map from only 96 images with a numerical aperture (NA)-matched annular illumination, and can be implemented simply by adding an LED array and polarization optics in a conventional optical microscope.

Several improvements can be considered in the reported platform. Dynamic imaging of optically anisotropic specimens is significant in various applications, such as cell dynamics^61^, contractile activity analysis of beating cardiomyocytes^62^, and liquid crystal monitoring^63^. However, the PS-IDT system developed in this study requires a relatively long acquisition time because of the use of a divergent LED light source and polarization optics. The LEDs with a 120° divergence angle were positioned at ~47 mm to generate a quasi-plane wave illumination, and thus the amount of light intensity at the sample plane was very small. A dome-shaped LED with built-in collimators^13^ or a combination of lasers and beam scanners (e.g., galvanometric scanners) can be considered for improving the light throughput, although the system may become bulky and introduce coherent speckle noise for lasers. In terms of the light loss due to polarized optics, polarization detection modules consisting of a polarizing beam splitter^62^ and multiple cameras can be employed. Another method to improve the imaging speed is to reduce the number of measurements. This approach reduces the total acquisition time as well as enables an efficient use of the memory and processing resources. In ref. ^15^, the tomographic scalar refractive index map of dynamic *C*. *elegans* were successfully obtained using only four LED annular illuminations, although it compromised the reconstruction image quality. Integration with more sophisticated reconstruction strategies, such as deep learning and deep image priors, may also be explored to realize high-speed 3D PSI without compromising the image quality. Indeed, several recent studies have demonstrated a highly effective deep-learning-aided tomographic reconstruction with limited angle illuminations^64,65^.

In the forward model of this demonstration, we assumed the PS-IDT prototype is an aberration-free system. However, practical polarization-sensitive imaging system may have optical aberrations including polarization-independent (e.g., defocus, astigmatism) and polarization-dependent aberrations induced by the birefringence of optics and interfaces^66,67^. These optical aberrations can degrade the reconstruction performance by breaking the consistency between the measured and estimated information. Recently, Dai et al., demonstrated vectorial Fourier ptychography reconstruction along with aberrated pupil matrix recovery by extending scalar aberration correction (embedded pupil recovery) without additional measurements^31^. Applying this approach, aberration-corrected PS-IDT reconstruction can be achieved by introducing an additional ptychographic update process into back-propagation to obtain both object spectrum and aberration pupil matrix in the Fourier plane.

The presented PS-IDT produces 3D anisotropy map of an object using vectorial MSBP forward model derived with Jones matrix in paraxial approximation, which considers only the in-plane optic axis in each layer. For biological applications involving flat or thin specimens (e.g., sectioned tissue slides or cell layers), this model may provide an efficient and accurate measurement. However, for applications that require a complete analysis of 3D anisotropic information, including the out-of-plane optic-axis orientation, our method must be refined to describe the complete dielectric tensor. Moreover, for high resolution (or high-NA) imaging of multiple scattering objects, our method should be refined to consider non-paraxial multiple scattering model. Recently, SSNP model was introduced as the forward model in scalar diffraction tomography^52,53^, and demonstrated its improved reconstruction performance for a non-paraxial multiple-scattering model. Shin et al. also demonstrated a holography-based tomographic 3 × 3 dielectric tensor imaging scheme for biaxial materials^41^. To realize the 3 × 3 tomographic measurement of dielectric tensors, additional measurements with a slightly tilted illumination were performed. The integration of these experimental and algorithmic strategies into the PS-IDT platform would enable a highly viable QPSI imaging platform for high resolution 3 × 3 tensor reconstruction for both weakly and multiple scattering objects.

The MSBP-based reconstruction framework requires a high computational power to compute the propagation operator at every layer in the forward and backward directions. In addition, PS-IDT, which handles four elements of the Jones matrix, performs numerically four times more computations than the scalar MSBP-based reconstruction method. This limitation can be addressed by leveraging the recent advances in parallel processing, achieved via GPU acceleration or cloud computing, to reduce the reconstruction time significantly.

Finally, we believe that PS-IDT can serve as a versatile, multi-modal imaging platform to provide a variety of structural and functional information about a 3D object. For example, combining this technique with computational fluorescence microscopy can provide the 3D distribution of labeled species along with the isotropic/anisotropic information^14^, and can also be utilized for fluorescence polarization analysis to reveal the protein orientations^68^ and structures of the cellular organelles^69^. In addition, 3D spectral anisotropy analysis can be performed by using either multi-color LEDs or hyperspectral image sensors, which will allow us to conduct various quantitative studies of, for example, 3D mapping and localization of plasmonic nanostructures based on their distinctive spectral and polarization signatures^70–72^.

## Methods

### Experimental setup

The PS-IDT prototype was constructed by implementing an LED microscopy with polarization optics for 3D PSI (Fig. 1c). As a light source, an annular LED array (central wavelength λ = 520 nm, 1586, Adafruit, USA) was used for the annular plane wave illuminations at various azimuthal angles. Recently, several researchers have shown that annular illuminations with the incident angle matching the objective NA ($NA_{obj}$) is the optimal condition for tomographic imaging^15,73–75^. This illumination scheme optimally encodes both low- and high-spatial frequency information across the entire 3D volume using a small number of intensity measurements. An annular LED array featuring a radius of 35 mm with 24 LEDs equally spaced in the azimuthal direction was used. To match the objective NA and illumination NA ($NA_{ill}$), the LED array was placed at a distance of 47 mm from the sample plane, and its center was carefully positioned on the optical axis. The distance (*h*), i.e., the separation between the LED array and the sample, was calculated by the following relation:

$$h \cong \sqrt {\frac{{r^2}}{{NA_{obj}^2}} - r^2}$$

where *r* is the radius of the annular LED array.

A 3D object was illuminated with a unique illumination angle by sequentially operating the individual elements of the LED array. A rotating wheel (LCFW5, Thorlabs, USA) equipped with left- and right-handed circular polarizers (CP42HE and CP42HER, Edmund Optics, UK) was placed between the sample plane and the annular LED array, and the circular polarizer was manually changed to modulate the input polarization state. After the interaction with the 3D anisotropic sample, the diffracted light was collected by a 0.6 NA, ×40 objective lens (CFI S Plan Fluor ELWD ADM 40XC, Nikon, Japan), and the tube lens (ACT508-200-A, Thorlabs, USA) formed the image on a scientific CMOS camera (pco.edge 4.2, PCO, USA). To acquire an intensity vector containing two orthogonally polarized intensity components, a motorized rotating linear polarizer (LPVISA100-MP2 and PRM1Z8, Thorlabs, USA) was placed in front of the image sensor. The angle of the absorption axis of the linear polarizer was aligned in the horizontal direction of the observation coordinates. The PS-IDT exhibited an axial resolution of 2.89 μm and a lateral resolution of 0.54 μm with an FOV of 80 μm × 80 μm.

### Data acquisition

For each polarizer/analyzer configuration, 24 raw intensity images were obtained by sequentially illuminating the LED elements. The LED array was driven by Arduino Uno, and all the images were acquired by a scientific CMOS camera (pco.edge 4.2, PCO, USA), which was synchronized with the LED source through an external trigger channel. Under the four polarizer/analyzer configurations, a total of 96 images were acquired for the PS-IDT reconstruction. The exposure time of each measurement was 1.5 s, resulting in a total acquisition time of approximately 144 s. Note that a relatively long exposure time was required because of the use of a divergent LED and polarization optics.

For calibrating the LED brightness, all the LED elements were sequentially turned on without the polarization optics (circular polarizer and linear polarizer in the illumination and detection paths, respectively) and sample, and 24 images were captured with an exposure time 0.3 s. After obtaining the brightness of all the LEDs by averaging the pixel intensities in the FOV, we used the information to normalize the intensity of the raw images in the subsequent measurements.

### Reconstruction procedures of PS-IDT

The reconstruction algorithm of PS-IDT is based on an iterative approach that involves gradient descent and back-propagation, along with the vectorial MSBP model. The reconstruction procedure is summarized as below:

1. Initialize the Jones matrix at each voxel in the reconstruction volume with an arbitrary 2 × 2 matrix. In our implementation, an identity matrix *I* was set as the initial Jones matrix. Then, initialize the iteration index *i* and the input polarization state index *m* as 0.
2. Increment the iteration index, $i \leftarrow i + 1$, and initialize the cost function, ${{{\mathrm{c}}}}\left( i \right) = 0$.
3. Increment the input polarization state, $m \leftarrow m + 1$, and initialize the illumination angle index, $\ell = 0$.
4. Increment the illumination angle index, $\ell \leftarrow \ell + 1$, and compute the incident electric field vector $$\vec E_{m,0}^\ell \left( {{{\boldsymbol{r}}}} \right) = \exp \left( {j{{{\boldsymbol{k}}}}_0^\ell \cdot {{{\boldsymbol{r}}}}} \right)\left[ {\begin{array}{*{20}{c}} 1 & {\left( { - 1} \right)^mj} \end{array}} \right]^T$$. Note that an even or odd *m* corresponds to an illumination with left- or right-handed circular polarization.
5. Using the incident electric field vector and estimated Jones matrix, evaluate the electric field vectors $\left\{ {\vec E_{m,n}^\ell \left( {{{\boldsymbol{r}}}} \right)|n = 1,2, \ldots ,N} \right\}$ at each layer of the reconstruction volume, and estimate the measurement amplitude $\left( {\left| {\boldsymbol{\mathcal{G}}_m^\ell \left\{ {\bar O\left( {{{{\boldsymbol{r}}}}_{3D}} \right)} \right\}} \right|} \right)$ using the vectorial MSBP forward model.
6. Using the corresponding intensity measurement $\vec I_m^\ell$, update the cost function for the current iteration as ${{{\mathrm{c}}}}\left( i \right) \leftarrow {{{\mathrm{c}}}}\left( i \right) + \mathop {\sum}\nolimits_{{{\boldsymbol{r}}}} {\left\| {\sqrt {\vec I_m^\ell \left( {{{\boldsymbol{r}}}} \right)} - \left| {\boldsymbol{\mathcal{G}}_m^\ell \left\{ {\bar O\left( {{{{\boldsymbol{r}}}}_{3D}} \right)} \right\}} \right|} \right\|_2^2}$, and initialize a residual vector, $\vec {\Psi}_m^\ell \left( {{{\boldsymbol{r}}}} \right),$ as
  7 $$\vec {\Psi}_m^\ell \left( {{{\boldsymbol{r}}}} \right) = {\boldsymbol{\mathcal{G}}}_m^\ell \left\{ {\bar O\left( {{{{\boldsymbol{r}}}}_{3D}} \right)} \right\} - diag\left( {\frac{{\boldsymbol{\mathcal{G}}_m^\ell \left\{ {\bar O\left( {{{{\boldsymbol{r}}}}_{3D}} \right)} \right\}}}{{\left| {\boldsymbol{\mathcal{G}}_m^\ell \left\{ {\bar O\left( {{{{\boldsymbol{r}}}}_{3D}} \right)} \right\}} \right|}}} \right)\sqrt {\vec I_m^\ell \left( {{{\boldsymbol{r}}}} \right)}$$
  where $diag\left( {\vec x} \right)$ is a diagonal matrix with the elements of the vector $\vec x$ as the diagonal elements. Note that matrix division, square root, and modulus are element-wise operations. Then, by substituting the residual vector into the inverse operation of Eq. 3, the back-propagation vector of the last layer of the reconstruction volume, $\vec {\Psi}_{m,N}^\ell \left( {{{\boldsymbol{r}}}} \right)$, is obtained.
7. The sample Jones matrix for each layer is updated based on the gradient descent and back-propagation from the last layer to the first layer using
  8 $$\vec O_n\left( {{{\boldsymbol{r}}}} \right) \leftarrow \vec O_n\left( {{{\boldsymbol{r}}}} \right) + diag\left( {\vec \alpha } \right)\vec E_{m,n}^{\ell ,{\dagger} }\left( {{{\boldsymbol{r}}}} \right)\bar H_{{\Delta}z}^{\dagger} \vec {\varPsi}_{m,n}^\ell \left( {{{\boldsymbol{r}}}} \right)$$
  where $\vec {\varPsi}_{m,n}^\ell \left( {{{\boldsymbol{r}}}} \right) = \bar O_k^{\dagger} \left( {{{\boldsymbol{r}}}} \right)\bar H_{{\Delta}z}^{\dagger} \vec {\varPsi}_{m,n + 1}^\ell \left( {{{\boldsymbol{r}}}} \right)$, and $\vec \alpha$ is the step-size vector. Note that $\vec O_n\left( {{{\boldsymbol{r}}}} \right)$ is the vector representation of the sample Jones matrix $\bar O_n\left( {{{\boldsymbol{r}}}} \right)$, i.e.,$$\vec O_n\left( {{{\boldsymbol{r}}}} \right) = \left[ {\begin{array}{*{20}{c}} {O_{n,xx}} & {O_{n,xy}} & {O_{n,yx}} & {O_{n,yy}} \end{array}} \right]^T$$. The details of the gradient computation is presented in Supplementary Information (Section 1).
8. Repeat steps (4)–(7) for each illumination angle.
9. Repeat steps (3)–(8) for each input polarization state.
10. Implement a proximal operation of 3D total variation^76^ and fast iterative shrinkage-thresholding algorithm^77^ on the reconstructed 3D Jones matrix to promote stability and increase the convergence speed.
11. Repeat (2)–(10) until a self-consistent solution is achieved. After reaching convergence, anisotropy properties, such as the mean phase delay ($\tilde \phi$), retardance (δ), and azimuthal optic-axis orientation (φ), are extracted from the reconstructed 3D Jones matrix using matrix diagonalization (Fig. 1d). Details on the extraction of the isotropic and anisotropic properties are provided in the “Methods” section.

### Calibration of illumination angles

PS-IDT requires an accurate knowledge of the illumination angles for robust 3D Jones matrix reconstruction. However, due to manufacturing imperfection or experimental misalignment of the LED array, the illumination angles may differ from the pre-defined angles in the experiment, which may cause significant reconstruction artifacts^78,79^. To mitigate the mismatch between the implementation and numerical model, we performed a self-calibration procedure before the reconstruction. In the demonstration, we implemented the algorithmic self-calibration method^78^ originally developed to correct the LED position in Fourier ptychography. This technique, which can quickly calculate the illumination angle from the raw data without additional measurements, has been utilized in various computational microscopy techinques^13,30,60^ as well as in FP. In Supplementary Information section 6, a comparison of the PS-IDT reconstruction performance before and after the calibration is provided.

### Extraction of the isotropic and anisotropic properties

Using the reconstructed Jones matrix information, the mean phase, retardance, and optic-axis orientation can be obtained through eigen analysis. The 3 × 3 Jones matrix of a non-depolarizing uniaxial material can be expressed as:

9 $$\bar O_{3 \times 3} = \bar R_\varphi ^{ - 1}\bar R_\theta ^{ - 1}\left[ {\begin{array}{*{20}{c}} {e^{j\phi _s}} & 0 & 0 \\ 0 & {e^{j\phi _s}} & 0 \\ 0 & 0 & {e^{j\phi _f}} \end{array}} \right]\bar R_\theta \bar R_\varphi$$

where $\bar R_\varphi$ and $\bar R_\theta$ are the coordinate rotation matrices for the azimuthal (*φ*) and polar angles (*θ*), respectively, and *ϕ*_s_ and *ϕ*_f_ are the phase delays of the slow and fast axes, respectively. Because Eq. 9 is symmetric, the optic-axis orientations (*φ* and *θ*) and phase delays (*ϕ*_*s*_ and *ϕ*_*f*_) can be easily obtained from the 3 × 3 Jones matrix by taking the eigenvalues and eigenvectors via diagonalization^22,34,80,81^. Yet, the reconstructed 2 × 2 Jones matrix is symmetric, there are three parameters that determine the birefringence in each voxel in PS-IDT. Therefore, the extraction of the anisotropic properties is an underdetermined problem. As a result, through the eigen analysis of the reconstructed Jones matrix, the fast-axis term ($e^{j\phi _f}$) and optic-axis orientation of the *xy*-plane (*φ*) can be directly extracted, but the slow-axis term ($e^{j\phi _s}$) and polar angle (*θ*) are coupled as

10 $$e^{j\phi _s^{Approx}} = e^{j\phi _s}\sin ^2\theta + e^{j\phi _f}\cos ^2\theta$$

where $\phi _s^{Approx}$ is the slow-axis phase term measured using the reconstructed 2 × 2 Jones matrix. As shown in Eq. 10, when the polar angle of the object’s optic axis decreases, the error in the extracted slow-axis phase term cannot be ignored. We performed a numerical error analysis on the estimated phase delay in the slow axis as a function of the optic axis polar angle, and found that the error is smaller than 10% for a polar angle larger than 68°. In our analysis, the mean phase ($\tilde \phi$) and retardance (δ) were defined as $\tilde \phi = {\textstyle{{\phi _s^{Approx} + \phi _f} \over 2}}$ and $\delta = \phi _s^{Approx} - \phi _f$, respectively, and were used to present the anisotropic information.

### Sample preparations

#### Potato starch granules slide

In this study, 1 mg of potato starch (100%) and 50 μL of the index matching oil (*n*_*oil*_ = 1.516, Cargille Laboratories, USA) were carefully mixed with a vortex mixer (model VM-10, Witeg Labortechnik, Germany). Then, the mixed sample was mounted on a microscope slide and covered with a coverslip for the PS-IDT imaging.

#### Tardigrades

Live tardigrades (*Hypsibius exemplaris*, 133960, Carolina Biological, USA) fed with Chlorella algae (152069, Carolina Biological, USA) were first carefully extracted with a micropipette and placed on microscope slides. The sample was then mounted in Hoyer’s medium, which is widely used for mounting tardigrades for microscope imaging^82,83^. The sample was covered with a coverslip for imaging. We prepared Hoyer’s medium by following the recipe reported in ref. ^83^. In brief, 200 g of chloral hydrate (302-17-0, Sigma-Aldrich, USA) was added to 50 mL of distilled water (7732-18-5, Sigma-Aldrich, USA), and heated to 60° until dissolved. Then, 30 g of gum arabic (9000-01-5, Sigma-Aldrich, USA) was added to the solution, and covered, stirred, and heated overnight. Finally, 20 g of glycerin (56-81-5, Sigma-Aldrich, USA) was added to the mixture, and the solution was filtered through a blotting paper. Typically, the refractive index of Hoyer’s medium is ~1.48, which is similar to that of tardigrade organs. After sealing and waiting for ~3 min, the tardigrades were imaged using PS-IDT.

## Supplementary information

Supplementary information

3D-rendering of the reconstructed anisotropic cell phantom

3D-rendering of the reconstructed numerical liquid crystal phantom

3D-rendering of the reconstructed potato starch granules

Depth scanning of the reconstructed whole tardigrade

3D-rendering of the reconstructed whole tardigrade
