# Supplementary material for: Polarization-sensitive intensity diffraction tomography
Source: Light Sci Appl. 2023 May 18;12:124. doi: 10.1038/s41377-023-01151-0 (PMC10195819; doi:10.1038/s41377-023-01151-0)
Supplement: Supplementary file 1 — Supplementary information [file 41377_2023_1151_MOESM1_ESM.docx]

# Supplementary Information for

**Polarization-sensitive intensity diffraction tomography**

Seungri Song^1^, Jeongsoo Kim^1^, Taegyun Moon^1^, Baekcheon Seong^1^, Woovin Kim^1^, Chang-Hyuk Yoo^2^, Jun-Kyu Choi^2^, and Chulmin Joo^1,*^

*^1^Department of Mechanical Engineering, Yonsei University, Seoul, 03722, Republic of Korea*

*^2^Small Machines Company, Ltd., Seoul, 04808, Republic of Korea*

**Corresponding author:* [*cjoo@yonsei.ac.kr*](mailto:cjoo@yonsei.ac.kr)

# Contents

1. Derivation of the gradient of objective function with respect to Jones matrix

2. PS-IDT-based numerical reconstruction of a weakly scattering object

3. PS-IDT reconstruction of a polystyrene microbead

# 4. Representative PS-IDT images of a tardigrade

5. Effect of optic-axis polar orientation on anisotropy measurement

6. Illumination angle calibration for PS-IDT reconstruction

# Section 1. Derivation of the gradient of objective function with respect to Jones matrix

In the PS-IDT reconstruction, the object Jones matrix for each layer is recursively updated using the gradient descent method. In this section, we provide details of the gradient computation of the loss function with respect to the Jones matrix defined in Eq. 5 in the manuscript. We consider the measurement with an illumination angle $\mathcal{l}$ and input polarization state $m$, and drop the indices $\mathcal{l}$ and $m$ from the deviations for notational simplicity.

The data fidelity term ($\mathcal{D}$) in Eq. 5 can be re-written and expanded as

|  | $\mathcal{D}\left\{ \bar{O}\left( \boldsymbol{r}_{3D} \right) \right\}\boldsymbol{=}\left\Vert\sqrt{\vec{I}\left( \boldsymbol{r} \right)}-\left\vert\mathcal{G}\left\{ \bar{O}\left( \boldsymbol{r}_{3D} \right) \right\} \right\vert\right\Vert_{2}^{2}$ $=\sqrt{\vec{I}^{\dagger}\left( \boldsymbol{r} \right)}\sqrt{\vec{I}\left( \boldsymbol{r} \right)}+\left\vert\mathcal{G}^{\dagger}\left\{ \bar{O}\left( \boldsymbol{r}_{3D} \right) \right\} \right\vert\left\vert\mathcal{G}\left\{ \bar{O}\left( \boldsymbol{r}_{3D} \right) \right\} \right\vert-2\sqrt{\vec{I}^{\dagger}\left( \boldsymbol{r} \right)}\left\vert\mathcal{G}\left\{ \bar{O}\left( \boldsymbol{r}_{3D} \right) \right\} \right\vert$ | (S1) |
| --- | --- | --- |

where the superscript $\dagger$ denotes the conjugate transpose operator, and the square root and modulus are element-wise operations.

Our goal is to reconstruct the Jones matrix of the *n*-th layer, $\bar{O}_{n}$, of the object using gradient descent. Accordingly, we re-arrange the Jones matrix and introduce an object vector ($\vec{O}_{n}$) made up of the elements of the object Jones matrix $\bar{O}_{n}$as

|  | $\vec{O}_{n}=\left[ \begin{matrix} O_{n,xx} \\ O_{n,xy} \\ O_{n,yx} \\ O_{n,yy} \end{matrix} \right]$ | (S2) |
| --- | --- | --- |

Using this object vector, the gradient of the data fidelity term can be expressed as

|  | $\nabla_{\vec{O}}\mathcal{D}\left\{ \bar{O}\left( \boldsymbol{r}_{3D} \right) \right\}=\left[ \frac{\partial\mathcal{D}\left\{ \bar{O}\left( \boldsymbol{r}_{3D} \right) \right\}}{\partial\vec{O}\left( \boldsymbol{r}_{3D} \right)} \right]^{\dagger}$ | (S3) |
| --- | --- | --- |

By taking partial derivative and complex-conjugate transpose of$\mathcal{D}$, one can obtain the gradient of $\mathcal{D}\left\{ \bar{O}\left( \boldsymbol{r}_{3D} \right) \right\}$ with respect to $\vec{O}_{n}$as

|  | $\nabla_{\vec{O}}\mathcal{D}\left\{ \bar{O}\left( \boldsymbol{r}_{3D} \right) \right\}=\left[ \frac{\partial}{\partial\vec{O}}\mathcal{G}\left\{ \bar{O}\left( \boldsymbol{r}_{3D} \right) \right\} \right]^{\dagger}\left[ \mathcal{G}\left\{ \bar{O}\left( \boldsymbol{r}_{3D} \right) \right\}-diag\left( \frac{\mathcal{G}\left\{ \bar{O}\left( \boldsymbol{r}_{3D} \right) \right\}}{\left\vert\mathcal{G}\left\{ \bar{O}\left( \boldsymbol{r}_{3D} \right) \right\} \right\vert} \right)\sqrt{\vec{I}\left( \boldsymbol{r} \right)} \right]$ | (S4) |
| --- | --- | --- |

With the definition of the forward operation, i.e., $\mathcal{G}\left\{ \bar{O}\left( \boldsymbol{r}_{3D} \right) \right\}=\bar{F}^{\dagger}\bar{P}\left( \boldsymbol{k} \right)\bar{F}\bar{H}_{-N\Delta z/2}\vec{E}_{N}\left( \boldsymbol{r} \right)$ (See Eq. 3 in the main text), Eq. S4 can be written as

|  | $\nabla_{\vec{O}}\mathcal{D}\left\{ \bar{O}\left( \boldsymbol{r}_{3D} \right) \right\}=\left[ \frac{\partial}{\partial\vec{O}}\vec{E}_{N}\left( \boldsymbol{r} \right) \right]^{\dagger}\vec{S}_{N}\left( \boldsymbol{r} \right)$ | (S5) |
| --- | --- | --- |

where $\vec{S}_{N}\left( \boldsymbol{r} \right)=\bar{H}_{-{N\Delta z}/2}^{\dagger}F^{\dagger}\bar{P}^{\dagger}\left( \boldsymbol{k} \right)F\left[ \mathcal{G}\left\{ \bar{O}\left( \boldsymbol{r}_{3D} \right) \right\}-diag\left( \frac{\mathcal{G}\left\{ \bar{O}\left( \boldsymbol{r}_{3D} \right) \right\}}{\left| \mathcal{G}\left\{ \bar{O}\left( \boldsymbol{r}_{3D} \right) \right\} \right|} \right)\sqrt{\vec{I}\left( \boldsymbol{r} \right)} \right].$

Prior to calculating $\frac{\partial}{\partial\vec{O}}\vec{E}_{N}\left( \boldsymbol{r} \right)$ in Eq. S5, we write Eq. 1 (in the main text) as follows using the introduced object vector $\vec{O}_{n}$:

|  | $\vec{E}_{n}\left( \boldsymbol{r} \right)=\bar{H}_{\Delta z}\bar{E}_{n-1}\left( \boldsymbol{r} \right)\vec{O}_{n}\left( \boldsymbol{r} \right)$ | (S6) |
| --- | --- | --- |

where the electric field matrix is defined as

|  | $\bar{E}_{n}\left( \boldsymbol{r} \right)=\left[ \begin{matrix} E_{n,x}\left( \boldsymbol{r} \right) & 0 & E_{n,x}\left( \boldsymbol{r} \right) & 0 \\ 0 & E_{n,y}\left( \boldsymbol{r} \right) & 0 & E_{n,y}\left( \boldsymbol{r} \right) \end{matrix} \right]$ | (S7) |
| --- | --- | --- |

The recursive formula Eq. 1 (in the main text) and Eq. S6 allow us to obtain

|  | $\frac{\partial}{\partial\vec{O}}\vec{E}_{n}\left( \boldsymbol{r} \right)=\bar{H}_{\Delta z}\bar{E}_{n-1}\left( \boldsymbol{r} \right)\frac{\partial}{\partial\vec{O}}\vec{O}_{n}\left( \boldsymbol{r} \right)+\bar{H}_{\Delta z}\bar{O}_{n}\left( \boldsymbol{r} \right)\frac{\partial}{\partial\vec{O}}\vec{E}_{n-1}\left( \boldsymbol{r} \right)$ | (S8) |
| --- | --- | --- |

Using Eq. S8, we obtain the following equality:

|  | $\left[ \frac{\partial}{\partial\vec{O}}\vec{E}_{n}\left( \boldsymbol{r} \right) \right]^{\dagger}=\left[ \frac{\partial}{\partial\vec{O}}\vec{O}_{n}\left( \boldsymbol{r} \right) \right]^{\dagger}\bar{E}_{n-1}^{\dagger}\left( \boldsymbol{r} \right)\bar{H}_{\Delta z}^{\dagger}+\left[ \frac{\partial}{\partial\vec{O}}\vec{E}_{n-1}\left( \boldsymbol{r} \right) \right]^{\dagger}\bar{O}_{n}^{\dagger}\left( \boldsymbol{r} \right)\bar{H}_{\Delta z}^{\dagger}$ | (S9) |
| --- | --- | --- |

Based on the recursion of Eq. S9 and $\vec{S}_{n-1}\left( \boldsymbol{r} \right)\boldsymbol{=}\bar{O}_{n}^{\dagger}\left( \boldsymbol{r} \right)\bar{H}_{\Delta z}^{\dagger}\vec{S}_{n}\left( \boldsymbol{r} \right)$ with the boundary condition $\left[ \frac{\partial}{\partial\vec{O}}\vec{E}_{0}\left( \boldsymbol{r} \right) \right]^{\dagger}=0$, we can finally obtain the gradient $\nabla_{\vec{O}}\mathcal{D}\left\{ \bar{O}\left( \boldsymbol{r}_{3D} \right) \right\}$ as

|  | $\nabla_{\vec{O}}\mathcal{D}\left\{ \bar{O}\left( \boldsymbol{r}_{3D} \right) \right\}\mathbf{=}\sum_{n=1}^{N} \left[ \frac{\partial}{\partial\vec{O}}\vec{O}_{n}\left( \boldsymbol{r} \right) \right]^{\dagger}\bar{E}_{n-1}^{\dagger}\left( \boldsymbol{r} \right)\bar{H}_{\Delta z}^{\dagger}\left( \boldsymbol{k} \right)\vec{S}_{n}\left( \boldsymbol{r} \right)$ | (S10) |
| --- | --- | --- |

# Section 2. PS-IDT-based numerical reconstruction of a weakly scattering object

We numerically simulated the PS-IDT imaging of a weakly scattering anisotropic object by considering a uniaxial crystal sphere with its slow- ($n_{s}$) and fast- ($n_{f}$) axis refractive indices as 1.49, and 1.48, respectively, and a radially aligned optic axis. The refractive index of the surrounding medium was set to 1.475.

Fig. S1a,c show the lateral and axial cross-sectional ground-truth mean phase and anisotropy maps of the object, and Fig. S1b,d present the corresponding images obtained using PS-IDT. The figures in the first row in Fig. S1 correspond to the images in the xy-plane at the center of the 3D object, and those in the second and third rows are the images in the yz and xz sections along the orange and red dashed lines in Fig. S1a1. Although there are some artifacts in the central region of the reconstructed images, which arise from discontinuities in the optic-axis orientation at the center, comparisons between the ground-truth and PS-IDT results demonstrated a great correspondence. The 3D-rendered mean phase and anisotropy maps of the phantom are shown in Fig. S1e,f. A video clip of the 3D rendering results is also provided in Supplementary Video 2.

**
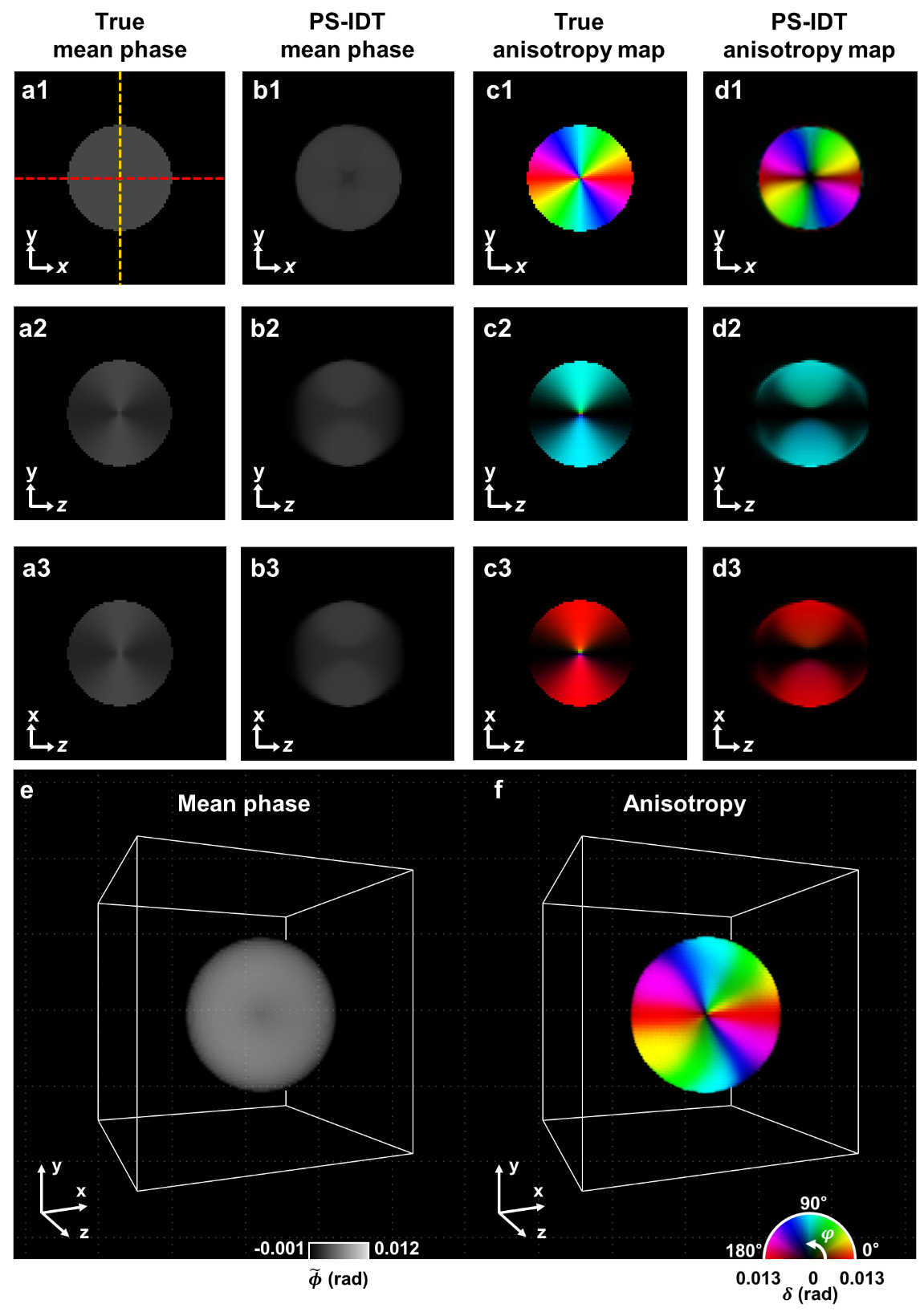
**

**Fig. S1** Numerical simulation result of a liquid crystal sphere, whose optical axis is radially aligned. **a1-3,c1-3** Ground-truth cross-sectional mean phase and anisotropy maps of the simulation object. **b1-3,d1-3** Corresponding cross-sectional mean phase and anisotropy maps obtained using PS-IDT. First row: images in the xy plane at the center of the 3D phantom; second and third rows: images in the yz and xz sections through the orange and red dashed lines in a1, respectively. **e,f** 3D-rendered images of the reconstructed mean phase and anisotropy information.

# Section 3. PS-IDT reconstruction of a polystyrene microbead


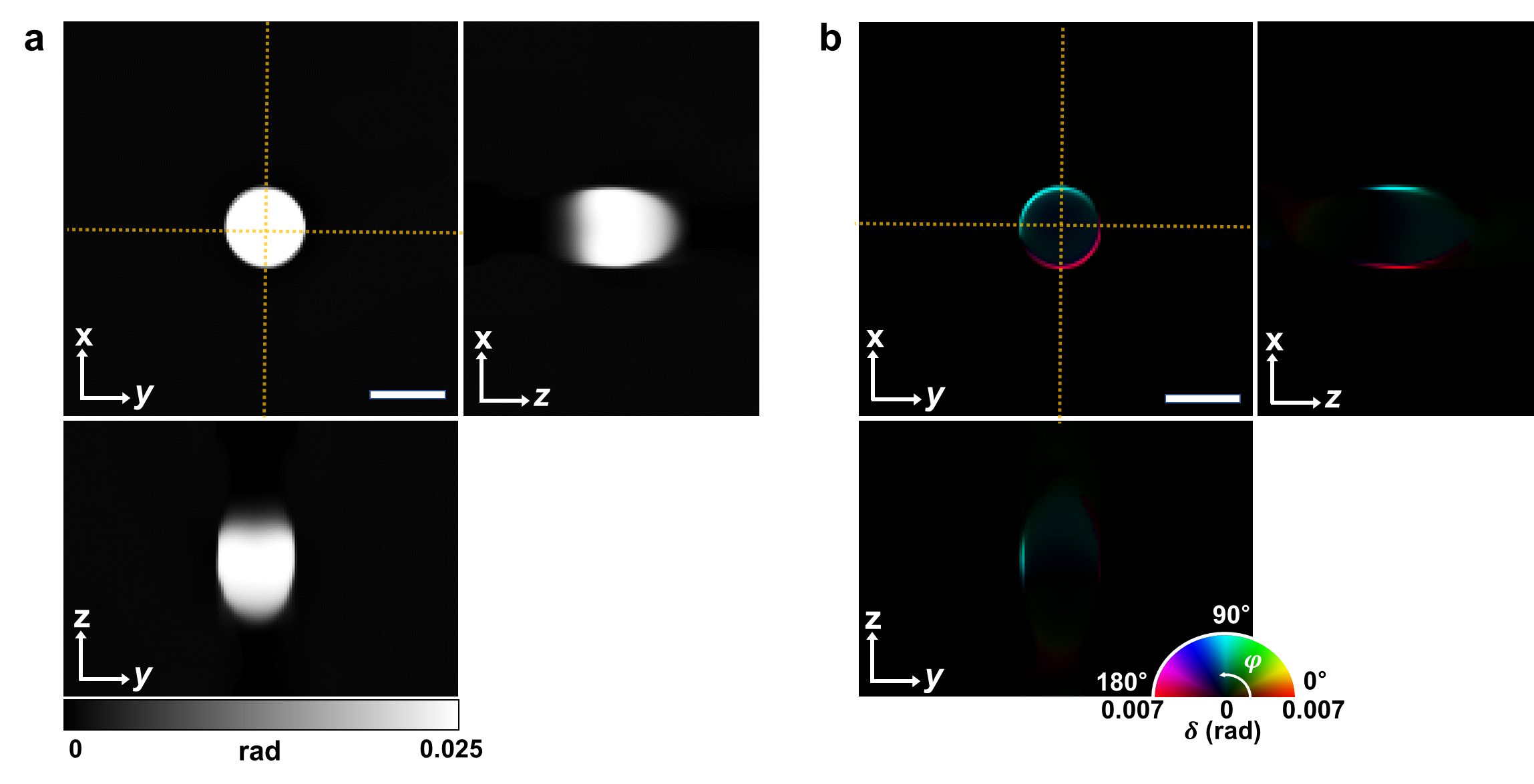


**Fig. S2** PS-IDT reconstruction of an optically isotropic $5 \mu m$ polystyrene microbead immersed in an immersion oil. **a** Reconstructed mean phase images of the xy, yz, and xz cross-sectional planes. **b** Reconstructed anisotropy maps of the xy, yz, and xz cross-sections. Scale bars: $5 \mu m$.

To validate the 3D polarization-sensitive imaging capability of PS-IDT, we first imaged a single 5-$\mu m$ polystyrene microbead ($n_{bead}=1.591$) immersed in an immersion oil with a refractive index of 1.58. For the PS-IDT reconstruction, the reconstruction volume was set as a stack of 100 layers with a spacing of $0.2 \mu m$ (i.e., $N=100$ and $\Delta z=0.2 \mu m$). Fig. S2a and S2b show the 3D mean phase and anisotropy maps, respectively, obtained from the reconstructed Jones matrix. The xz and yz cross-sectional images present the information in the plane along the orange dashed lines in the xy plane image. Because the polystyrene bead is optically transparent and isotropic, the phases in the slow and fast axes were found to be identical. Therefore, the mean phase was evenly measured (Fig. S2a), and no information was found inside the sphere in the anisotropy map (Fig. S2b). The measured mean phase of the polystyrene bead was 0.025 rad, which matches well with the estimated value of 0.027 based on the specification (W500CA, Thermo Fisher Scientific). The theoretical phase value was obtained as $\frac{2\pi}{\lambda}\left( n_{\mathrm{bead}}-n_{medium} \right)\Delta z$. Note that the ring-shaped anisotropy information was recovered at the bead edge as shown in Figure S2b. This anisotropic signal, known as the edge birefringence, is caused by the reflection and refraction of light at sharp transitions or discontinuities in the refractive index (optical boundaries). This phenomenon is typically observed in polarized images of transparent objects with sharp edges, such as crystals and microbeads^1-4^. Note that the edge birefringence is a consequence of the optical boundary itself, and not of the structural details in the vicinity of the boundary^1^.

# Section 4. Representative PS-IDT images of a tardigrade


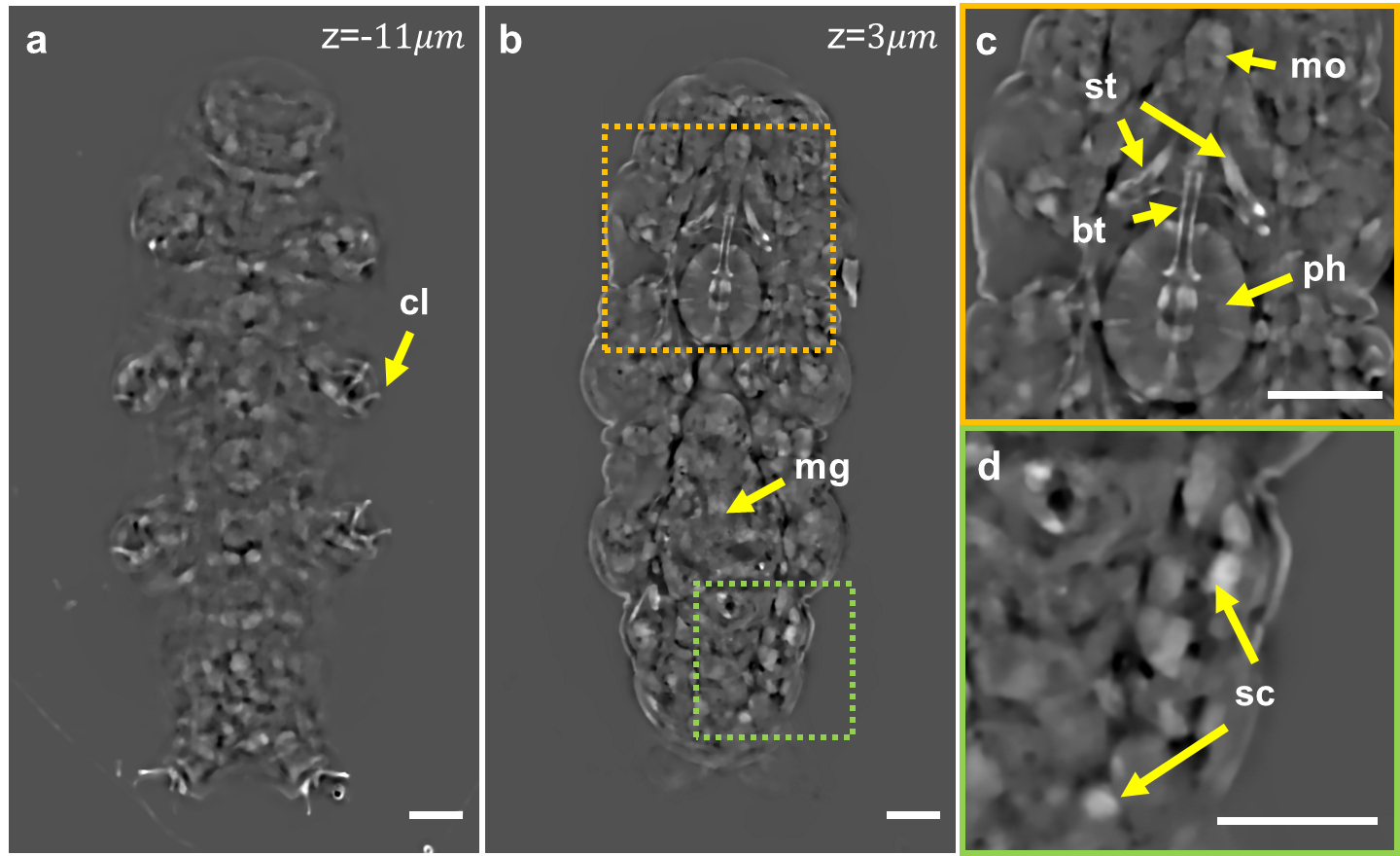


**Fig. S3** Representative mean phase images of a tardigrade reconstructed by PS-IDT. **a,b** Full field mean phase images of tardigrade at different depth sections. **c,d** Enlarged mean phase images for the regions marked as orange and green dashed square boxes. Abbreviations: bt, buccal tube; cl, claw; mg, midgut; mo, mouth; ph, pharynx; sc, storage cells; st, stylet. Scale bars: $8 \mu m$.

Due to their optical scattering property, the 3D structural analysis of tardigrade using optical microscopy has been challenging. PS-IDT enabled us to visualize and analyze the 3D structure of various organs of tardigrade in visible regime. Stylet and granules exhibiting optical anisotropy were analyzed in Fig. 4 of the main text, and only optically isotropic structures were analyzed in this section. Fig. S3 shows exemplary mean phase images in two layers where some distinct organs were well visualized. As shown in Fig. S3a, eight legs and claws (cl) were observed at z=-$11 \mu m$. In the layer corresponding to the center of tardigrade, the complex distribution of organs, including the feeding apparatus and midgut, was observed over the head and body (Fig. S3b-d). Fig. S3c and S3d show enlarged mean phase images in the regions marked as orange and green dashed square boxes in Fig. S3b, respectively. The structures of the stylet, buccal tube, mouth, and pharynx constituting the feeding apparatus were clearly visualized (Fig. S3c), and storage cells, which are free floating cells within the body cavity, were also observed in various parts of the body (Fig. S3d). Depth-resolved mean phase maps are provided in supplementary video S4.

# Section 5. Effect of optic-axis polar orientation on anisotropy measurement


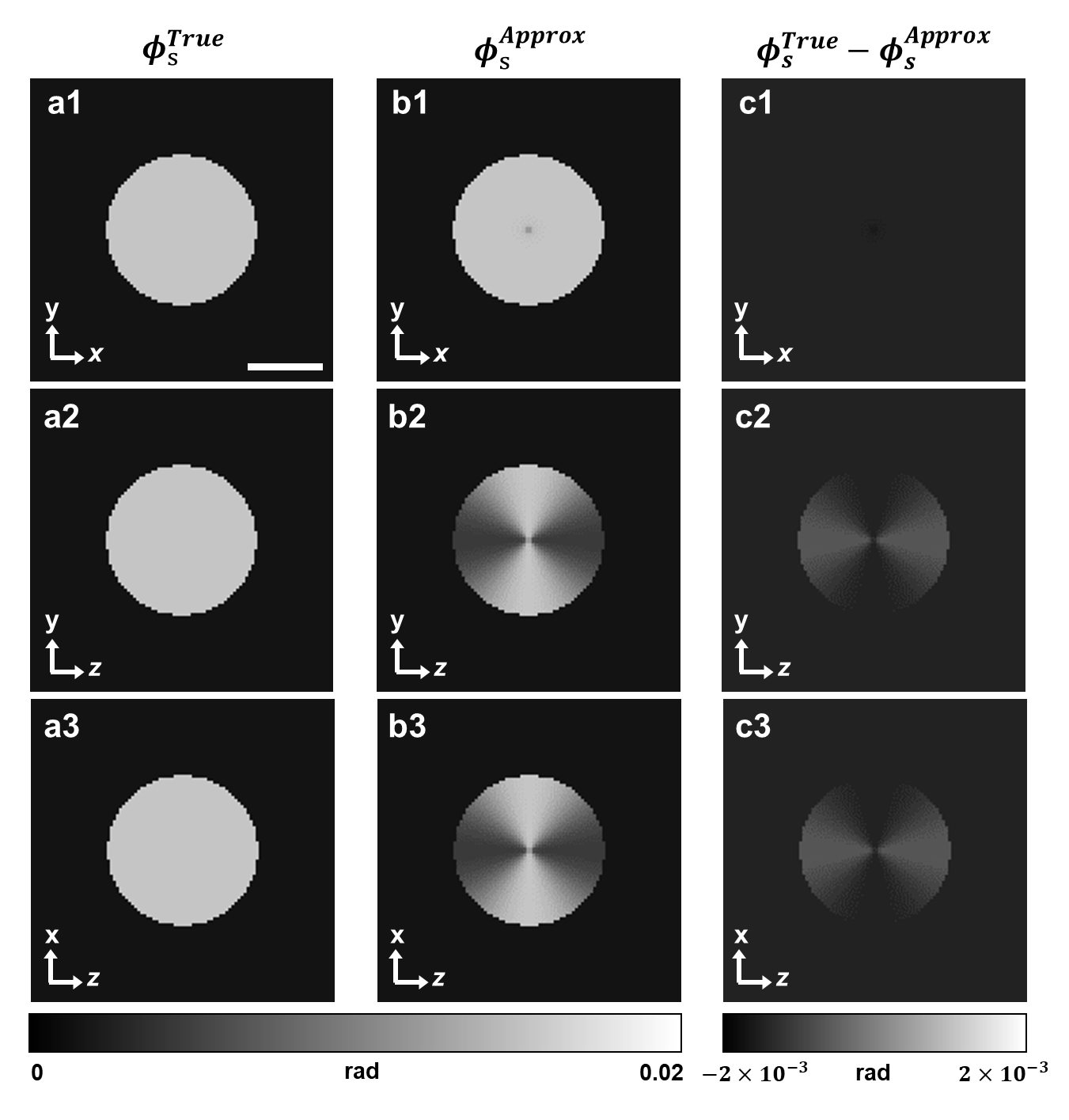


**Fig. S4** Comparison between the true and approximated slow-axis phase delay ($\phi_{s}$) of a uniaxial liquid crystal digital phantom. **a** True phase $\phi_{s}^{\mathrm{True}}$ of the xy, yz, and xz cross-sectional planes. **b** Approximated slow-axis phase $\phi_{s}^{\mathrm{Approx}}$ of the xy, yz, and xz cross-sections. **c** Differences between the true and approximated $\phi_{s}$. Scale bar: 4 $\mu m$.

In most 3D polarization-sensitive diffraction tomography techniques, including PS-IDT, only a partial anisotropy information of the sample can be recovered with a $2\times2$ Jones matrix^5,6^. While the $2\times2$ Jones matrix includes anisotropic phase delays ($\phi_{s}$ and $\phi_{f}$) and optical axis orientation ($\varphi$ and $\theta$) information of the sample, these four parameters cannot be completely determined, because the matrix is symmetric. In PS-IDT, $\phi_{f}$, $\phi_{s}^{Approx}$, and $\theta$ are extracted from the $2\times2$ Jones matrix through diagonalization (as described in the main manuscript). $\phi_{s}$ is, on the other hand, expressed as

|  | $\phi_{s}^{Approx}=\ln\left( e^{j\phi_{s}}{sin}^{2} \theta+e^{j\phi_{f}}{cos}^{2} \theta\right)$ | (S11) |
| --- | --- | --- |

When the optic axis of a uniaxial crystal is placed on the xy-plane (i.e., $\theta=90^{\circ}$), $\phi_{s}^{\mathrm{Approx}}$ is identical to $\phi_{s}$, and thus, the phase delay in the slow axis can be accurately measured. However, $\phi_{s}^{\mathrm{Approx}}$ deviates from the actual $\phi_{s}$, as the polar angle of the optical axis decreases. To evaluate the effect of the optic-axis polar angle on the measurement of $\phi_{s}$, we examined the estimation error in $\phi_{s}$ for the uniaxial liquid crystal sphere considered in Section 2. This object features a radially-directed optical axis distribution, and therefore can be used to evaluate the measurement error in $\phi_{s}$as a function of the optic-axis polar angle.

Fig. S4 shows the numerical comparison of the true and approximated $\phi_{s}$ in the lateral (first row) and axial cross-sections (second and third rows). The true $\phi_{s}$ is designed to have a uniform value in all the areas of the sphere (Fig. S4a). However, as shown in Fig. S4b,c, the error in the approximated $\phi_{s}$ increases as the polar angle approaches $0^{\circ}$ (perpendicular to xy-plane) in the axial cross-section. We quantitatively measured the error between the true and approximated $\phi_{s}$ as $\phi_{s}^{Error}=\frac{\phi_{s}^{True}-\phi_{s}^{Arrox}}{\phi_{s}^{True}}\times100$. For the liquid crystal phantom, $\phi_{s}^{Error}$ was found to be smaller than 10 % for $\theta$ >68°.


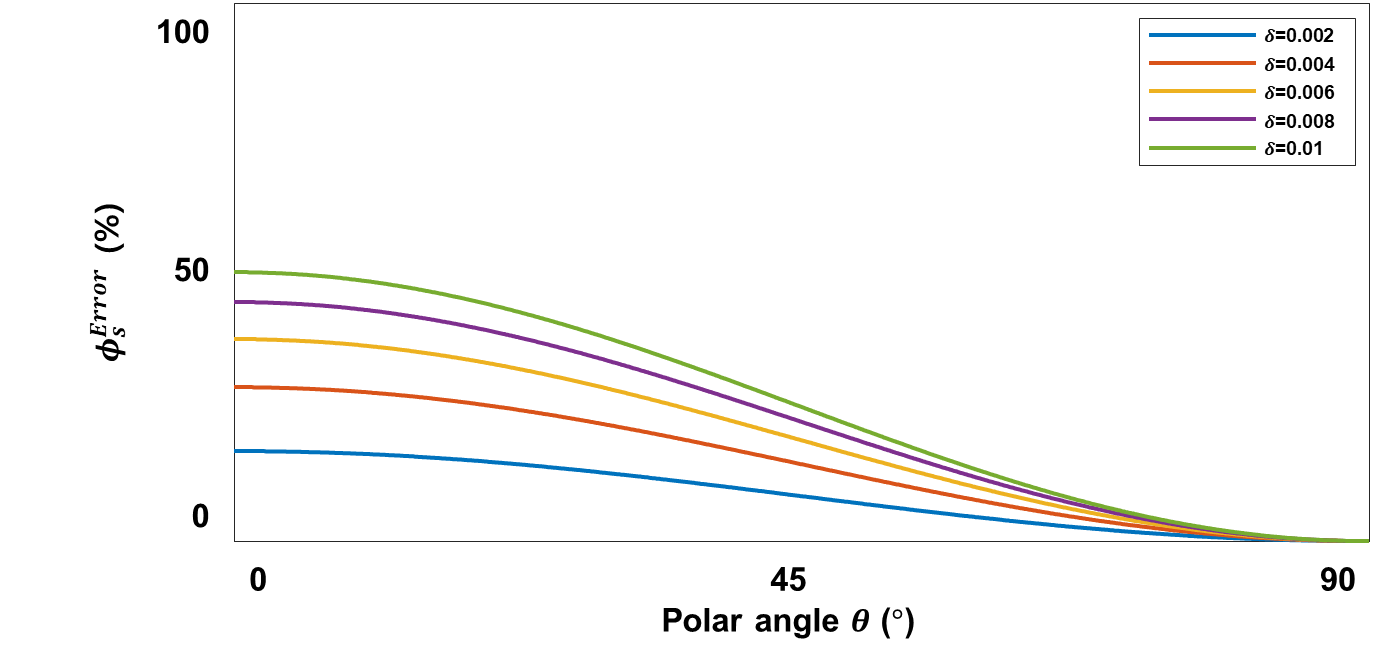


**Fig. S5** Measurement error of $\phi_{s}$ ($\phi_{s}^{Error}$) as a function of $\theta$ and $\delta$ of the object

Note that $\phi_{s}^{Error}$ depends on the polar angle of the optic axis ($\theta$) as well as the difference between $\phi_{s}$ and $\phi_{f}$, namely retardation $\delta$ (see Eq. (S11)). Fig. S5 shows $\phi_{s}^{Error}$ as a function of the optic-axis polar angle at different retardance values. One can see that the difference between the true and approximated $\phi_{s}$ is small for samples with small retardance. Indeed, many polarization imaging techniques assume that the optic axis lies in the xy-plane in optically anisotropic biological samples, which typically exhibit small birefringence^4,7^. In the future, we intend to extend our PS-IDT platform to incorporate $3\times3$ tensor reconstruction capabilities to measure all the anisotropic parameters, such as $\phi_{s}$, $\phi_{f}$, $\theta$, and $\varphi$.

# Section 6. Illumination angle calibration for PS-IDT reconstruction


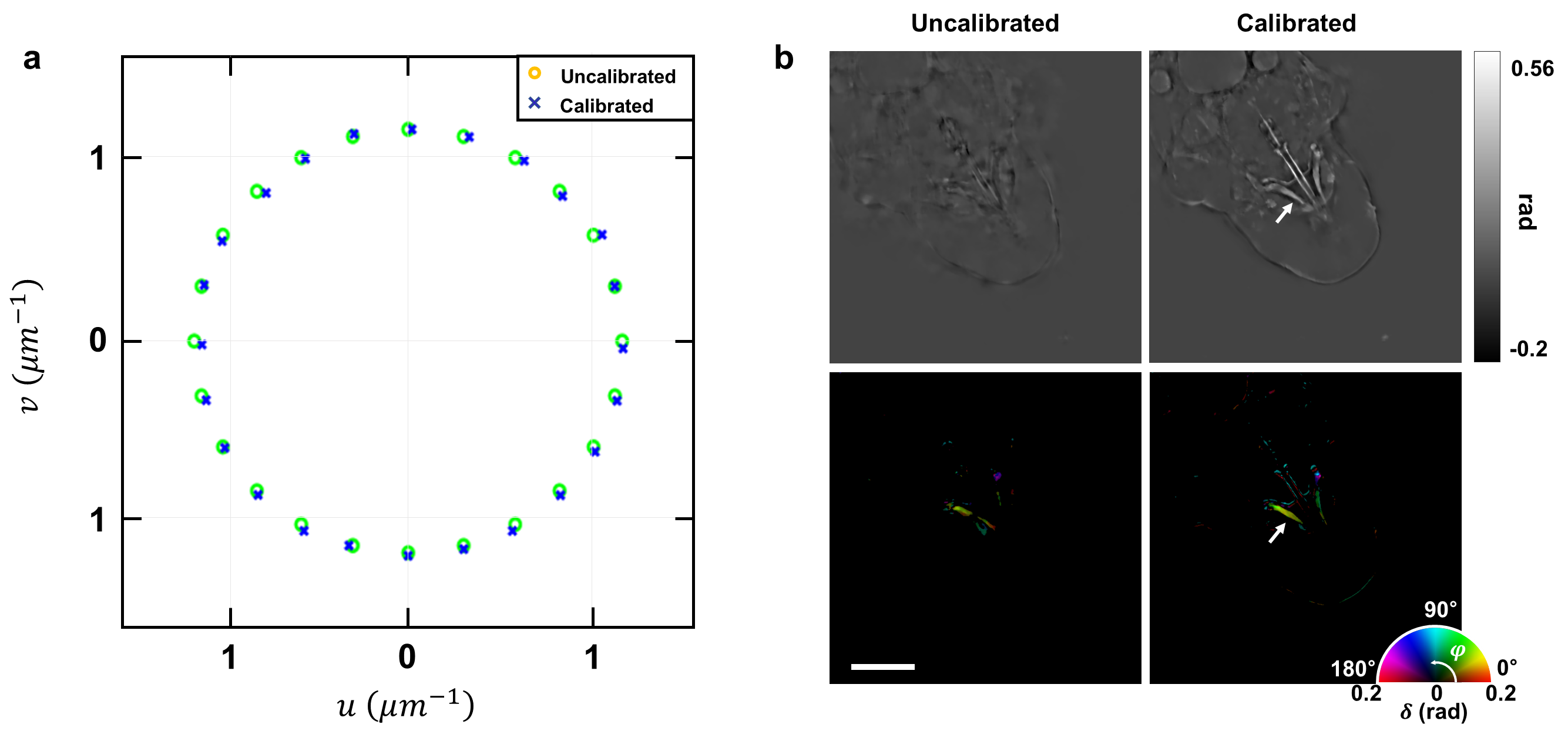


**Fig. S6** Calibration of the illumination angle for PS-IDT reconstruction. **a** Uncalibrated (green circle) and calibrated (blue cross) illumination angles plotted in the spatial frequency coordinates. **b** Comparison of the reconstructed mean phase and anisotropy maps of a tardigrade head at the center of the reconstruction volume before and after the angle calibration. The white arrow indicates the stylet. Scale bar: $20 \mu m$.

We employed a self-calibration algorithm that corrects for misalignment of the LED positions, enabling an accurate PS-IDT reconstruction. Fig. S6a shows the pre-defined (green circle) and calibrated (blue cross) illumination angles of the tardigrade measurements in the spatial frequency coordinates. The spatial coordinates $u$ and $v$ are defined as

|  | $u=\frac{{NA}_{obj}}{\lambda}\cos\varphi_{LED}$ | (S12) |
| --- | --- | --- |

|  | $v=\frac{{NA}_{obj}}{\lambda}\sin\varphi_{LED}$ | (S13) |
| --- | --- | --- |

where ${NA}_{obj}$ and $\varphi_{LED}$ are the numerical aperture of objective and the azimuth angle of the LEDs at the LED plane, respectively. Through the self-calibration method, the illumination angle error, which is difficult to adjust with manual alignment only, was corrected (Fig. S6a). Figure S6b highlights the improvement in the PS-IDT imaging performance through the illumination angle correction. Although the pre-defined and calibrated illumination angles did not differ significantly, the PS-IDT reconstruction without illumination angle correction failed to recover the sample information in both the mean phase and anisotropy images. In the images reconstructed with angle calibration, the structures of the tardigrade, including the stylet (white arrow), were clearly visible.

# References

1. Oldenbourg R. Analysis of edge birefringence. *Biophysical journal* 1991, **60**(3)**:** 629-641.

2. Yeh L-H, Ivanov IE, Guo S-M, Chhun BB, Hashemi E, Han MH*, et al.* uPTI: uniaxial permittivity tensor imaging of intrinsic density and anisotropy. Novel Techniques in Microscopy; 2021: Optica Publishing Group; 2021. p. NM3C. 4.

3. Dai X, Xu S, Yang X, Zhou KC, Glass C, Konda PC*, et al.* Quantitative Jones matrix imaging using vectorial Fourier ptychography. *Biomedical Optics Express* 2022, **13**(3)**:** 1457-1470.

4. Song S, Kim J, Hur S, Song J, Joo C. Large-area, high-resolution birefringence imaging with polarization-sensitive fourier ptychographic microscopy. *ACS Photonics* 2021, **8**(1)**:** 158-165.

5. Xu S, Dai X, Yang X, Zhou KC, Kim K, Pathak V*, et al.* Tensorial tomographic differential phase-contrast microscopy. *arXiv preprint arXiv:220411397* 2022.

6. Saba A, Lim J, Ayoub AB, Antoine EE, Psaltis D. Polarization-sensitive optical diffraction tomography. *Optica* 2021, **8**(3)**:** 402-408.

7. Götzinger E, Pircher M, Dejaco-Ruhswurm I, Kaminski S, Skorpik C, Hitzenberger CK. Imaging of birefringent properties of keratoconus corneas by polarization-sensitive optical coherence tomography. *Investigative ophthalmology & visual science* 2007, **48**(8)**:** 3551-3558.
